# The corpus callosum and creativity revisited

**DOI:** 10.3389/fnhum.2024.1443970

**Published:** 2024-09-12

**Authors:** Warren S. Brown, Lynn K. Paul

**Affiliations:** ^1^Travis Research Institute, Fuller School of Psychology & Marriage and Family Therapy, Pasadena, CA, United States; ^2^International Research Consortium for the Corpus Callosum and Cerebral Connectivity (IRC^5^), Pasadena, CA, United States; ^3^Division of Humanities and Social Sciences, California Institute of Technology, Pasadena, CA, United States

**Keywords:** corpus callosum, creativity, connectivity, split-brain, agenesis of the corpus callosum

## Abstract

In 1969 Joseph Bogen, a colleague of Roger Sperry and the neurosurgeon who performed commissurotomy on Sperry’s “split-brain” study participants, wrote an article subtitled “The Corpus Callosum and Creativity.” The article argued for the critical role of the corpus callosum and hemispheric specialization in creativity. Building on a four-stage model of creativity (learning, incubation, illumination, refinement) and Sperry’s innovative studies, the Bogens posited that in the intact brain, creativity relies on two opposing functions of the corpus callosum: (a) interhemispheric inhibition to facilitate simultaneous and independent activity of uniquely-specialized processing centers during *learning* and *incubation* and (b) interhemispheric facilitation to support the increased bi-hemispheric integration and coordination which produces *illumination.* This article revisits the Bogens’ theory considering scientific discoveries over the past 50 years. We begin by reviewing relevant findings from split-brain studies, and then briefly consider findings from studies that examine the association of creativity with callosal structure and function in neurotypical participants. Finally, we provide an in-depth discussion of creativity in persons with *agenesis of the corpus callosum* (ACC)—the congenital absence of the corpus callosum. These three lines of inquiry strongly support the theory suggested by Bogen and Bogen in 1969 and provide further clarification regarding the critical and unique role of the corpus callosum in creative cognition.

## 1 Introduction

*Creativity has not only made the human race unique in Nature; what is more important for the individual, it gives value and purpose to human existence.* Bogen and Bogen (1969).

In 1969 Joseph Bogen, the neurosurgeon who performed commissurotomy on the patients studied by Roger Sperry, wrote a noteworthy article entitled “The Other Side of the Brain III: The Corpus Callosum and Creativity” (Bogen and Bogen, 1969). The paper, co-authored with his artist wife, argued for the critical role of the corpus callosum (CC) and hemispheric specialization in creative thought. Support for this theory originally came from Sperry’s paradigm-shifting findings regarding hemispheric lateralization of cognitive functions as evident in commissurotomy patients (Sperry, 1968, 1974, 1982). Studies of these so-called ‘split-brain’ patients also revealed that despite retaining many cognitive skills, severing the CC and other interhemispheric connections limited their capacity for fostering creative solutions to complex, novel problems and generating imaginative ideas (Hoppe, 1988).

This article revisits the Bogen and Bogen (1969, 1988) theory regarding the CC and creativity considering scientific discoveries over the past 50+ years. We begin by reviewing relevant findings from the split-brain studies, then consider findings from studies that examine the association between callosal structure/function and creativity in neurotypical participants. Finally, we provide an in-depth discussion of creativity in persons with *agenesis of the corpus callosum* (ACC)—the congenital absence of the CC, which some have referred to as a natural split-brain. These three lines of inquiry strongly support the Bogen and Bogen (1969) theory that creativity arises from the capacity to share information between the cerebral hemispheres, and presumably to integrate the outcomes of their distinct processing modes, as well as the increase in processing power and speed gained by richly linked cerebral hemispheres.

## 2 Studies of “split-brain” patients

The core outcome of commissurotomy has been named the “split-brain syndrome.” Bogen (1993) summarized the syndrome in terms of 4 basic symptoms/outcomes:

- *Social ordinariness*—The cognitive and functional weaknesses are not readily distinguishable in ordinary social situations, but only identified with sensitive neuropsychological instruments.
- *Lack of interhemispheric transfer*—Visual and tactile information originally processed in one hemisphere is not available to the other hemisphere. Consequently, each hemisphere can have independent percepts, memories and choices (e.g., Forster and Corballis, 2000; Corballis and Corballis, 2001).
- *Hemispheric specialization*—Commissurotomy provided a context for observing specialized processing in the separated hemispheres. For example, language responses were typically elicited for stimuli presented to the left hemisphere (but not to the right), and visual–spatial or emotional responses were more accurate for stimuli presented to the right hemisphere (reviewed in Sperry, 1974, 1982).
- *Compensatory phenomena*—Over post-surgical time, commissurotomy patients develop non-callosal interhemispheric sharing of information such that in most everyday experiences both hemispheres have the benefit of the same information (Campbell et al., 1981).

Deficiencies in creativity were not included among the core features of the split-brain syndrome, but rather were addressed as a likely consequence of these more obvious characteristics.

## 3 Theory of the corpus callosum and creativity

The Bogen and Bogen (1969, 1988) theory regarding the importance of the corpus callosum in creativity was heavily dependent on two aspects of the split-brain syndrome: lack of interhemispheric transfer and hemispheric specialization.

Among Sperry’s most noteworthy findings regarding creativity was the consistent demonstration of right hemisphere involvement in spatial analysis, facial processing, and a social–emotional awareness. Based on split-brain patients, as well as those with right hemisphere brain damage, Sperry (1982) argued that the right hemisphere is specialized for what he termed “spatial and imagistic” processes (p. 1225), such as perception of non-verbal sounds, including musical chords, and various forms of visuo-spatial construction and analysis. While the experience and expression of emotion is bilateral, the analysis of the contextual, social, and personal significance of emotional events is most robust in the right hemisphere (e.g., Van Lancker, 1991; Schwartz et al., 1975). This constellation of capacities led to a generalization that creativity and artistic expression are produced primarily from independent right hemisphere activity. However, as argued by Zaidel (2013), this assumption is not supported by any robust scientific evidence, but rather it is primarily promoted by popular media.

Building on Wallas’s (1926) four-stage model of creativity (learning, incubation, illumination, refinement), Sperry’s innovative studies, and the growing literature on the specialization of the right hemisphere, Bogen and Bogen (1969, 1988) posited that creativity relies on two opposing functions of the CC at specific stages of the creative process. First, during the *learning* and *incubation* phases, interhemispheric inhibition via the callosum facilitates simultaneous and independent activity of differently specialized processing centers in left and right cerebral hemispheres which promote a wider variety of cognitive perspectives. Second, interhemispheric facilitation via the CC supports a period of increased bi-hemispheric integration and coordination which produces *illumination*. Thus, in the intact brain, callosally-mediated interhemispheric interactions, both inhibitory and facilitatory, are critical to aspects of creative cognition.

Early results from studies of persons with commissurotomy gave preliminary, though often merely suggestive, support to the CC theory of creativity. For example, based on psychoanalytic interviews of 12 commissurotomy patients, Hoppe (1988, 1989) reported a limited use of cognitive displacement and symbolization, as well as restricted (“unimaginative,” “utilitarian”) fantasy life suggestive of limitations in elaborative and creative thought. TenHouten et al. (1985, 1986) presented commissurotomy and neurotypical participants with a short film that implicitly symbolized loss and death through use of music (no dialog), after which they were asked to provide their general impressions, write a short paragraph about the movie, answer questions about the symbolic content, and talk about feelings elicited by the film. Responses from the commissurotomy group were generally more concrete and included fewer affect words than responses from the comparison group. They also did not fantasize about or interpret the significance of symbols within the film, suggesting limited capacity to creatively elaborate beyond concrete interpretation of the images and music.

## 4 The neuroscience of creativity

Since the Bogen and Bogen publication in 1969, contemporary neuroscience has further illuminated the CC and creativity theory by demonstrating associations between various tests of creativity and CC structure in neurotypical individuals. For example, inductive reasoning performance (i.e., convergent thinking) has been positively correlated with CC mid-body size (Jung et al., 2014; Jung et al., 2010a; Jung et al., 2010b) and divergent thinking has been negatively correlated with overall CC size (but not with the volume of other white matter structures, Moore et al., 2009). Diffusion tensor imaging studies have reported CC structural integrity (factional anisotropy) is positively correlated with verbal but not visual creativity (Wu et al., 2021) and is positively correlated specifically with divergent thinking (Takeuchi et al., 2010).

## 5 The natural split brain

Agenesis of the corpus callosum (ACC) involves the congenital absence of the CC, either complete or partial (Jinkins et al., 1989; Paul et al., 2007). ACC results from a disruption of neural development during the 7th to 20th embryonic weeks (Edwards et al., 2014) which prevents some, or all, of the approximately 190 million corpus callosum axons from crossing between the left and right hemispheres. ACC occurs in at least 1 in 4000 births, making it one of the more commonly occurring congenital brain disorders (Glass et al., 2008; Wang et al., 2004).

Some have referred to ACC as a ‘natural split-brain’, but that disregards important distinguishing features of ACC. First, unlike the split-brain surgery that severs all interhemispheric connections, ACC typically does not involve a full-anatomic disconnection of the cerebral hemispheres. Most importantly, in ACC the anterior commissure (a much smaller cerebral commissure connecting right and left fronto-temporal regions) is typically present and functional (Jinkins et al., 1989; Mancuso et al., 2019). Second, split-brain surgery is conducted after the brain is completely formed, but ACC occurs during ongoing brain development, when there is opportunity for greater structural and functional compensation. Structurally, this may include enlargement of the anterior commissure (Hetts et al., 2006) or development of atypical interhemispheric connections (Tovar-Moll et al., 2007). Functionally, we have documented intact-bilateral resting state networks in individuals with complete ACC (Tyszka et al., 2011), but greater interhemispheric transfer of sensory information than commissurotomy patients (albeit weaker than neurotypical controls, Paul et al., 2007). Additionally, there is preliminary evidence of reduced language laterality in adults with complete-primary ACC (Hinkley et al., 2016; Nair et al., 2013). Thus, while ACC is not structurally or functionally equivalent to a ‘split-brain’, congenital absence of callosal connections does compromise cognitive functioning and possibly cortical organization.

Over the past 25 years, we have examined a broad range of cognitive and psychosocial functioning in individuals for whom ACC is the *primary* brain abnormality affecting cognitive and psychosocial outcomes (i.e., individuals with complete ACC, no other brain abnormalities visible on clinical MRI, and general intelligence within average range). Despite broadly average cognitive functioning, we have identified a relatively consistent pattern of mild to moderate cognitive deficiencies in individuals with primary ACC: (1) deficiencies in interhemispheric transfer of perceptual and motor information, (2) slowed cognitive processing speed, and (3) difficulties in complex novel problem-solving (Brown and Paul, 2019). We posited that most of the wider pattern of cognitive and psychosocial deficiencies reported in ACC are secondary to (i.e., can be explained as outcomes of) these 3 core aspects of the syndrome. However, the theory of creativity suggested by Bogen and Bogen (1969) raises the possibility that creativity may also be a compromised core cognitive process in ACC.

## 6 Core cognitive limitations of ACC

The fundamental cognitive impact of ACC is reduced (but not eliminated) capacity for interhemispheric transfer (and inhibition) of sensory and motor information. Indeed, in adults with complete ACC, we have shown that there is compromised capacity to accurately match two visual stimuli flashed simultaneously in the right and left visual fields (Brown and Jeeves, 1993; Brown et al., 1999), limited transfer of tactile information from one hand to the other (Dunn et al., 2000), and reduced interhemispheric coordination of bilateral hand movements (Mueller et al., 2009). However, these studies also demonstrate compensation for callosal absence in people with ACC. For example, individuals with ACC (but not a split-brain patient) performed normally in bilateral matching of two letters (upper or lower case “A” or “B”), but performance fell to chance when attempting to match more complex stimuli (patterns comprised of 6 dots). Similarly, participants with ACC had the bilateral motor information necessary to coordinate hand movements, but the interactions were less timely and efficient (Mueller et al., 2009), perhaps due to inadequate interhemispheric inhibition supporting independent motor control.

These results suggest a limited degree of interhemispheric interaction is taking place in ACC. As expected, given the absence of a commissural pathway the size of the CC, interhemispheric transfer is limited to small amounts of data…perhaps only what can be encoded into fewer bits of information and transferred over subcortical pathways or via the anterior commissure.

Limited capacity for long-range coordination between cortical regions and reallocation of networks to support interhemispheric transfer may also account, in large part, for the second core symptom: slowed cognitive processing (Marco et al., 2012). However, the question of this essay is, given the hypothesized relationship between the CC and creativity proposed by Bogen and Bogen (1969), what are the implications of these significant limitations on interhemispheric interactions for the manifestation of creativity in persons with ACC?

## 7 Problem solving in ACC

Elaborative thinking, imagination and creativity are critical to the ability to solve complex novel problems—the third core limitation in primary ACC (Brown and Paul, 2019). That is, relative to neurotypical controls, adults with ACC display increasing difficulty imagining solutions to problems, particularly as cognitive demands increase.

With respect to interhemispheric interactions during problem-solving in neurotypical individuals, research has shown that bi-hemispheric networks are active during cognitively complex problem-solving (Koivisto, 2000; Reuter-Lorenz and Stanczak, 2000; Weissman and Banich, 2000). However, reliance on interhemispheric networks decreased with continued practice in a particular problem-solving domain (Cherbuin and Brinkman, 2005; Maertens and Pollmann, 2005; Weissman and Banich, 2000; Weissman and Compton, 2003). This suggests the corpus callosum is particularly important for complex and novel (un-practiced) problem-solving.

Consistent with the implications of this research, persons with primary ACC typically perform within the normal range on over-learned, well-practiced, cognitive processes that are presumably less dependent on interhemispheric coordination (i.e., crystallized intelligence), but exhibit difficulties on more novel, less practiced, cognitive tasks (i.e., fluid intelligence). This pattern is illustrated by intact performance on most verbal and spatial portions of standardized intelligence scales and tests of basic academic skills, such as single-word reading, spelling, and basic math calculation, paired with significant difficulties on cognitive tasks, such as math reasoning, concept formation, and novel complex problem-solving, (i.e., fluid intelligence; Brown et al., 2012; Erickson et al., 2013; Schieffer et al., 2000). The impact of task complexity in primary ACC is evident in comparison of Full-Scale Intelligence Quotient (an index of crystallized intelligence) and performance on the Raven’s Progressive Matrices (an index of fluid intelligence; Raven, 1940; Raven et al., 2003). While performance on the basic (standard) Raven’s version was consistent with FSIQ, performance on the more complex (advanced) Raven’s test fell significantly below FSIQ (Schieffer et al., 2000).

The capacity for imaginative inference is a critical aspect of problem solving in the social domain. Comprehension of complex social situations involves inferences regarding the mental states and intentions of others, the likely nature of preceding events, and the contextual meanings of non-literal statements (such as sarcasm, metaphoric speech, and uncommon idioms). Using social situations presented in short videos vignettes, Symington et al. (2010) showed that persons with ACC were deficient in the recognition of emotions, understanding paradoxical sarcasm, and interpreting conversational social cues. When asked to think through potential responses to complex interpersonal scenarios, individuals with ACC had great difficulty imagining the impact of potential actions on others and their likely emotional consequences, and performance declined in response to greater situational complexity (Young et al., 2019). Responses to this task generally had non-typical semantic content, including the use of fewer words with emotion and cognitive content (insight). Similarly, persons with ACC exhibit limited knowledge of social norms and judgments of what is appropriate to do in different contexts and tend to over adhere to norms without recognizing contexts in which norms can be appropriately violated (Brown et al., 2020).

These studies of social problem solving in ACC suggest a basic deficiency in the capacity to imagine and infer social information not immediately apparent in the current context, particularly when demands for correct inferences and appropriate imagination are greatest.

## 8 Imagination and elaborative processing in ACC

As we have argued, adequate problem-solving demands a degree of elaborative thinking and imagination. In this light, we recently completed two critical studies examining capacity for imagination and elaboration in individuals with primary ACC, specifically addressing the ability to imagine and infer a social narrative from restricted visual prompts. In one study (Renteria-Vazquez et al., 2021) participants were asked to describe eight short video animations involving 2 triangles moving about in a manner suggestive of interpersonal interactions (Castelli et al., 2000, 2002). Neurotypical individuals typically describe these animations using interpersonal inferences involving imagined intentions, cognitions, and emotions attributed to the actions of the two triangles (Castelli et al., 2002). In the other study (Brown et al., 2024), participants were asked to generate stories in response to six socially evocative pictures from the Thematic Apperception Test (TAT) (Murray, 1943). Stories were to include a beginning, middle, and end, as well as what the characters were thinking and feeling.

Narratives responses from both studies were analyzed using Topic Modeling, or Latent Dirichlet Allocation (LDA) (Atkins et al., 2012; Blei, 2012; Blei and Lafferty, 2006; Liu et al., 2016). LDA is a process that identifies the semantic core of a text. Within topic modeling, degree of similarity or difference between the topic models of two texts is indicated by a perplexity score. Perplexity is defined as a “canonical measure of goodness [of fit] that is used in language modeling to measure the likelihood of held-out data to be generated from the underlying (learned) distributions of the model” (AlSumait et al., 2008, p. 6). A higher perplexity value indicates deviation from the reference text, while a lower perplexity indicates similarity. For each item in both studies, perplexity was used to measure the relationship between the topic model of a single participant’s response (ACC and neurotypical) and the topic model of the common core response derived from the concatenation of the responses of all (other) neurotypical participants. In both studies, perplexity values indicated greater semantic variation among responses from neurotypical controls than the responses of persons with ACC, both with respect to overall mean perplexity scores (*η_p_*^2^ = 0.573 for animations, Renteria-Vazquez et al., 2021; *η_p_*^2^ = 0.588 for responses to the TAT pictures, Brown et al., 2024), and for each animation or TAT picture analyzed separately. In other words, semantic content produced by participants with ACC was primarily restricted to the commonly identified information (core response) and neurotypical individuals provided richer narratives based on imaginative elaboration beyond the stimulus content. Additional analysis of TAT responses indicates the deficiencies in imaginative elaboration evident in adults with ACC are specific to (or most pronounced with respect to) cognitive, emotional and interpersonal content.

## 9 Conclusion

Evidence of limited capacity for imagination, inference, elaborative thought, and creativity of persons with ACC supports the Bogen and Bogen (1969, 1988) hypothesis regarding the contribution of interhemispheric inhibitory and facilitatory activation to creativity and underscores the importance of the corpus callosum. Limitations in creativity evident after commissurotomy (severing of all interhemispheric commissures), are also evident when disconnection is restricted to the corpus callosum and occurs within the course of fetal development. In primary ACC, residual inter-hemispheric connections such as the anterior commissure and recruitment of compensatory cortical processing systems support a limited degree bi-hemispheric coordination and information transfer but cannot fully support the complex large-scale integration and coordination of hemisphere-specific processes subserved by the corpus callosum. In the future, studies of hemispheric specialization in ACC may provide additional clarification regarding callosal contributions to creativity. However, current advances in characterizing the consequences of ACC affirm the opinion of Bremer (1956) as quoted by the Bogens in 1988 (p. 299), ‘the corpus callosum subserves “the highest and most elaborate activities of the brain” – in a word, creativity.’

## Data Availability

The original contributions presented in the study are included in the article/supplementary material, further inquiries can be directed to the corresponding author.
